# Visually and Tactually Guided Grasps Lead to Different Neuronal Activity in Non-human Primates

**DOI:** 10.3389/fnins.2021.679910

**Published:** 2021-07-19

**Authors:** Daniela Buchwald, Hansjörg Scherberger

**Affiliations:** ^1^Neurobiology Laboratory, Deutsches Primatenzentrum GmbH, Göttingen, Germany; ^2^Faculty of Biology and Psychology, University of Goettingen, Göttingen, Germany

**Keywords:** grasping, object interaction, non-human primate, multi-sensory, electrophysiology

## Abstract

Movements are defining characteristics of all behaviors. Animals walk around, move their eyes to explore the world or touch structures to learn more about them. So far we only have some basic understanding of how the brain generates movements, especially when we want to understand how different areas of the brain interact with each other. In this study we investigated the influence of sensory object information on grasp planning in four different brain areas involved in vision, touch, movement planning, and movement generation in the parietal, somatosensory, premotor and motor cortex. We trained one monkey to grasp objects that he either saw or touched beforehand while continuously recording neural spiking activity with chronically implanted floating multi-electrode arrays. The animal was instructed to sit in the dark and either look at a shortly illuminated object or reach out and explore the object with his hand in the dark before lifting it up. In a first analysis we confirmed that the animal not only memorizes the object in both tasks, but also applies an object-specific grip type, independent of the sensory modality. In the neuronal population, we found a significant difference in the number of tuned units for sensory modalities during grasp planning that persisted into grasp execution. These differences were sufficient to enable a classifier to decode the object and sensory modality in a single trial exclusively from neural population activity. These results give valuable insights in how different brain areas contribute to the preparation of grasp movement and how different sensory streams can lead to distinct neural activity while still resulting in the same action execution.

## 1. Introduction

Sensory-motor transformation flexibly links sensory information from several sensory modalities to meaningful action activations. Our different senses constantly pick up information about our environment, which needs to be processed, interpreted, and ultimately results in various actions (Gibson, 1958; Schlegel et al., 2009; Schaffelhofer and Scherberger, 2016). One of the most important senses for many animals is vision, but other senses like touch or proprioception are equally relevant, in particular for object manipulation (Land and Fernald, 1992; Grigg, 1994; Goodman and Bensmaia, 2018). Van Essen estimated 2003 that in humans about 27% of cortex is dedicated to process predominantly visual input, while only 7% are dedicated to predominantly somatosensory and motor processes (Van Essen, 2003).

Since grasping movements are so abundant in primates, they make a good example to investigate how the primate brain generates movements and how different senses can influence these movements (Raos et al., 2004; Stone and Gonzalez, 2014; Iturrate et al., 2016; Camponogara and Volcic, 2019). Each grasping movement is a joint effort of many different brain areas. For example if an object is seen, visual information is processed through visual areas like the primary visual cortex V1 or the anterior intraparietal area AIP, where it might serve as a basis to select future actions (Snowden et al., 1991; Murata et al., 2000; Lehmann and Scherberger, 2015; Schaffelhofer and Scherberger, 2016; Self et al., 2019). AIP is connected to its own specific areas (e.g., secondary somatosensory cortex, premotor cortex) and therefore plays an important role for processing visual and tactile information for the planning and execution of grasping movements (Binkofski et al., 1999; Luppino et al., 1999; Borra et al., 2007). Furthermore, past studies have shown that AIP responds to visual properties of objects that are about to be grasped. For example, Baumann et al. (2009) demonstrated that neurons in AIP encode object orientations as well as grip types during a delayed grasping task with a visually presented target. It is therefore an important area for the processing of object interactions (Taira et al., 1990; Sakata et al., 1995; Borra et al., 2007; Lehmann and Scherberger, 2013; Schaffelhofer, 2014; Schaffelhofer and Scherberger, 2016).

Not all objects are perceived purely visually. In fact, primates often touch new objects with their hands to gather additional, tactile object properties, and they are very efficient in doing so (Klatzky et al., 1993; Englerova et al., 2019). This tactile information is passing through multiple brain areas, including the primary somatosensory cortex, where object features and structures are processed (Warren et al., 1986; Johansson, 1991; Delhaye et al., 2018; Umeda et al., 2019; Liu et al., 2021). Such information can then be used to generate more targeted actions, and absence of haptic feedback can severely impair the control of hand movements (Miall et al., 2019; Okorokova et al., 2020). However, somatosensory cortex is not only processing tactile feedback but also proprioception, therefore providing also important feedback about the position and kinematics of the arm and hand during grasping (Grigg, 1994; Filimon et al., 2009; Delhaye et al., 2018; Chowdhury et al., 2020; Lutz and Bensmaia, 2021). For example, seeing a food item might trigger a grasping action, whereas seeing a predator might instead lead to a fleeing response. To coordinate the involved muscles of the body, the brain first needs to set an action goal and from there a movement plan, before the goal-directed movement can be executed. Two areas involved in these processes are the premotor and motor cortex, where movements are first planned and then executed (Fritsch and Hitzig, 1870; Churchland et al., 2006; Fluet et al., 2010; Arbuckle et al., 2020). The hand area in the premotor cortex, area F5, is directly and bi-directionally connected to AIP, and both areas are part of the fronto-parietal grasping network (Luppino et al., 1999; Rizzolatti and Luppino, 2001; Borra et al., 2007). Information from various senses serves not only as a basis for movement selection, but also provides important feedback to adjust ongoing movements and therefore suggests the involvement of various sensory brain areas for motor control (Saunders and Knill, 2004; Christensen et al., 2007; Oya et al., 2020).

In this study we investigated how the brain generates grasping movements based exclusively on visual or tactile object information. In order to do so, we not only tapped into the fronto-parietal grasping network of premotor area F5, AIP and M1, but also added parallel recordings from S1 in order to obtain a fuller picture of how different sensory modalities influence the sensory and motor side of grasp planning and execution.

## 2. Materials and Methods

### 2.1. Animals

Animal housing and all experiments were performed in accordance with European and German law and in agreement with the “Guidelines for the Care and Use of Mammals in Neuroscience and Behavioural Research” (National Research Council, 2003), as well as the NC3Rs “Guidelines for non-human primate accommodation, care and use” (National Centre for the Replacement, Refinement and Reduction of Animals in Research, 2017). Authorization for conducting this experiment was granted by the Animal Welfare Division of the Office for Consumer Protection and Food Safety of the State of Lower Saxony, Germany (permit no. 14/1442 and 19/3132).

For this project we trained one purpose-bred, male rhesus monkey (*Macaca mulatta*) in a delayed grasping task. He was born in 2011 at the German Primate Center (Deutsches Primatenzentrum GmbH, Göttingen, Germany) and housed together with another monkey in a setting that additionally allowed visual interaction with other groups of monkeys. On training days, intake of fluids through water bottles and rewards (such as juice or fruits) was monitored, since fluids served as the main reward for the animal. The animal was conditioned using positive reinforcement training, in which correct actions always resulted in a reward for the animal. Access to food was never restricted.

### 2.2. Implantation and Neuronal Signal Acquisition

In order to observe brain activity while the monkey explores and lifts up objects, a titanium head post was implanted on the skull in a sterile procedure and eight floating microelectrode arrays (FMA, Microprobes for Life Sciences, Gaithersburg, MD, USA, see Musallam et al., 2007) were implanted in cortex several months later in a second procedure. Each array consisted of 36 electrodes, where 32 were used to record brain activity and 4 served either as ground or reference electrodes. The length of the electrodes varied from 1.5 to 7.1 mm. For further details on the implantation methods, see (Michaels et al., 2015; Buchwald, 2020).

Since the goal of this study was to investigate visual and tactile object recognition, grasp planning, and finally grasp execution, four brain areas were chosen for FMA implantation: anterior intraparietal cortex (AIP), primary somatosensory cortex (S1, area 3b), primary motor cortex (M1), and premotor cortex (area F5). To sample from a larger number of channels from each area, each cortical area was implanted with two arrays.

After the animal fully recovered (about 10–14 days post-op), the implants were connected to two 128-channel neural signal processors (Cerebus systems, Blackrock Microsystems Inc., Salt Lake City, UT, USA) that were synchronized according to manufacturer instructions, which allowed for data acquisition from all 256 electrodes in parallel. Data was recorded with a sampling rate of 30 kHz and 16 bit resolution, and all neural data was stored on a hard drive together with behavioral data for offline analysis (see Analysis methods, below). Behavioral data was recorded using various sensors on the setup, as described in the following section.

### 2.3. Experimental Setup

During animal training and recording sessions, the monkey was comfortably seated in a custom made primate chair that was adjusted to the monkey's size. His head was fixed using the implanted titanium head post to ensure that the cables of the recording system were not moving during recording, and a reward system for fluid rewards was positioned in front of the animal's mouth.

#### 2.3.1. Turntable Setup

**Figure 1:**
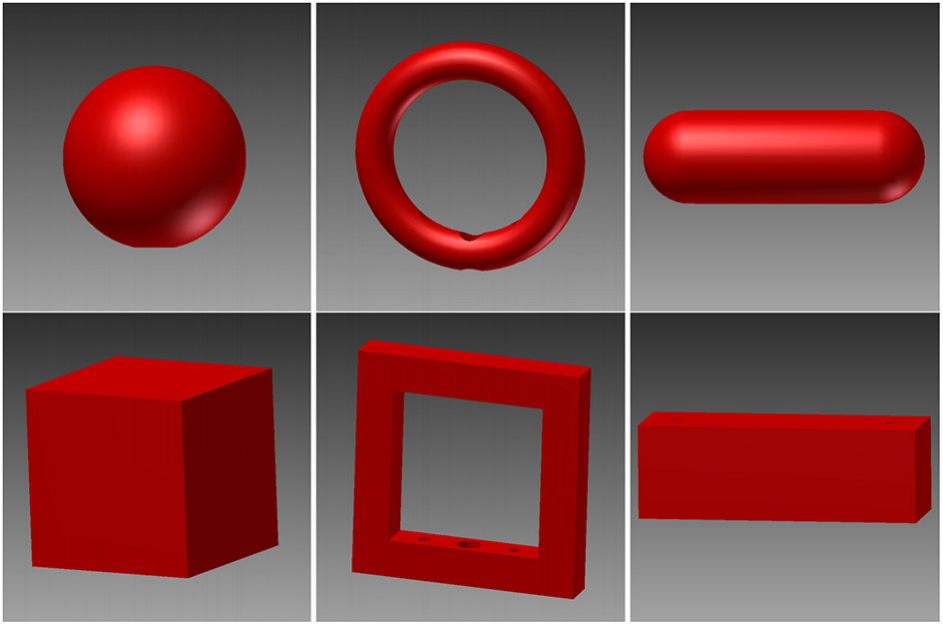
Illustration of the six objects used in this study. Six objects were designed to encourage different grasps and to contain distinct visible and tactile features. Top: sphere, ring, and rounded bar. Bottom: cube, edged version of the ring, and box.

**Figure 2:**
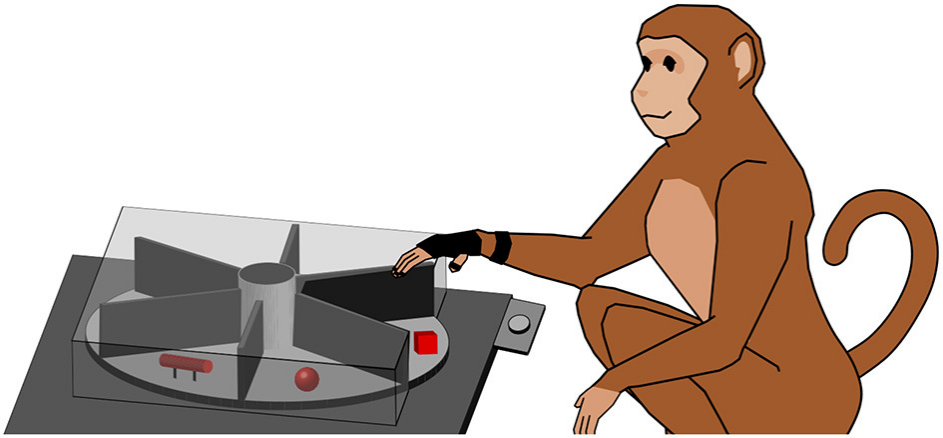
Turntable setup. The monkey working on the turntable (simplified). The turntable consists of a plate (light gray) that features six different objects (red). The object in front of the animal has to be lifted, while the other ones are out of view (illustrated by a cover). For hand tracking, the animal wears a (black) data glove. A handrest button (light gray) is located between animal and turntable.

To explore how objects are grasped, a variety of different objects was needed and we therefore employed a turntable setup, similar to those described in previous studies (Schaffelhofer, 2014; Schaffelhofer et al., 2015; Schaffelhofer and Scherberger, 2016). In this setup, a round object plate that fits six objects is operated by a motor. This way each object can be moved to the front in random order without human interaction. Objects were designed to have similar size and equal weight (120 g, including object and connected counter-weight below the plate) to keep the lifting effort similar between objects. Objects were 3D printed in plastic (PA 2200; Electro Optical Systems GmbH, Munich, Germany) by Shapeways Inc. (New York, USA). Objects are displayed in Figure 1. In order to allow only one object to be visible and reachable during each trial, several walls out of black plastic were mounted in the setup, see Figure 2 for illustration.

To instruct the animal to reach out and touch or grasp the object, a red LED was projected onto the object, using a half-transparent mirror. This avoided any unwanted illumination of the object, since the light source was far away from the object and only appeared to be located on top of the object by superposition from the mirror. To keep track of the behavior of the monkey, different sensors were used: a light barrier detected when the object was lifted, and a handrest button detected when the animal's hand was resting on it. Data from these sensors were recorded with a real-time data acquisition system (National Instruments Corp., Austin, TX, USA) and stored to disk together with the neural data. A more detailed description of the setup used in this study can be found in Buchwald et al. (2021).

#### 2.3.2. Magnetic Data Glove

**Figure 3:**
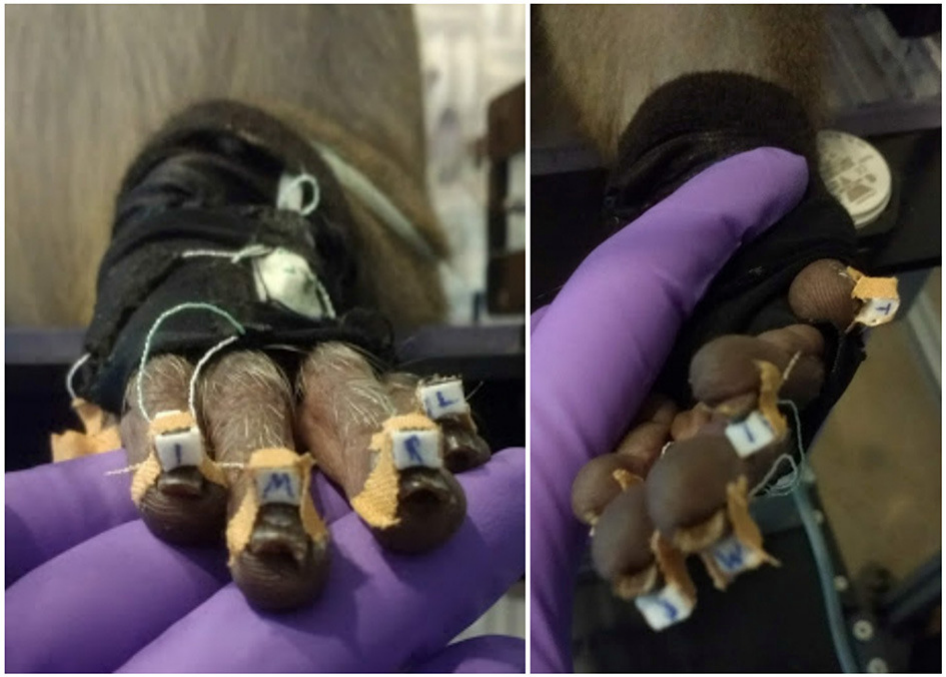
Magnetic data glove. Individual sensor coils (white boxes) are fixed to the finger nails using super glue and Leucotape, taking care not to cover the finger tips. Additional two sensors on the dorsum of the hand and the wrist are sawn onto a custom made glove that the animal was trained to wear. Purple: gloved hand of the experimenter.

Hand movements of the animal were tracked with a custom-built, magnetic data glove (see Schaffelhofer and Scherberger, 2012). This approach was extremely robust against occlusions, since no line of sight was required for hand tracking, in contrast to many visual tracking methods; the animal's hand simply had to be positioned within the magnetic field to get reliable data. The glove itself consisted of seven sensors on the finger nails of the monkey, and the dorsum and the wrist of the hand. To secure the sensor position on the finger nails, appropriate super glue and Leucotape were used, paying attention that the finger tips of the animal were not covered by the tape to allow free finger tip sensation (see Figure 3). The remaining two sensors were sewed to a custom made glove that was adjusted to the animal's hand. The magnetic field generator (Wave, Northern Digital Inc., Waterloo, Canada) was placed below the object plate. The sampling rate of the data glove was 70–100 Hz (depending on computer load). Hand kinematics were stored on a separate computer and synchronized with the neural data by sending a synchronization signal to the neural signal processor that was stored along with the neural data.

### 2.4. Behavioral Paradigm

To instruct grasping movements with various sensory object information, the monkey was trained in a delayed-grasping task. He was instructed to first either look at an object or to explore it with his hand in the dark before quickly grasping and lifting it up. This way the monkey only had either visual or tactile information to base his grasping action on.

**Figure 4:**
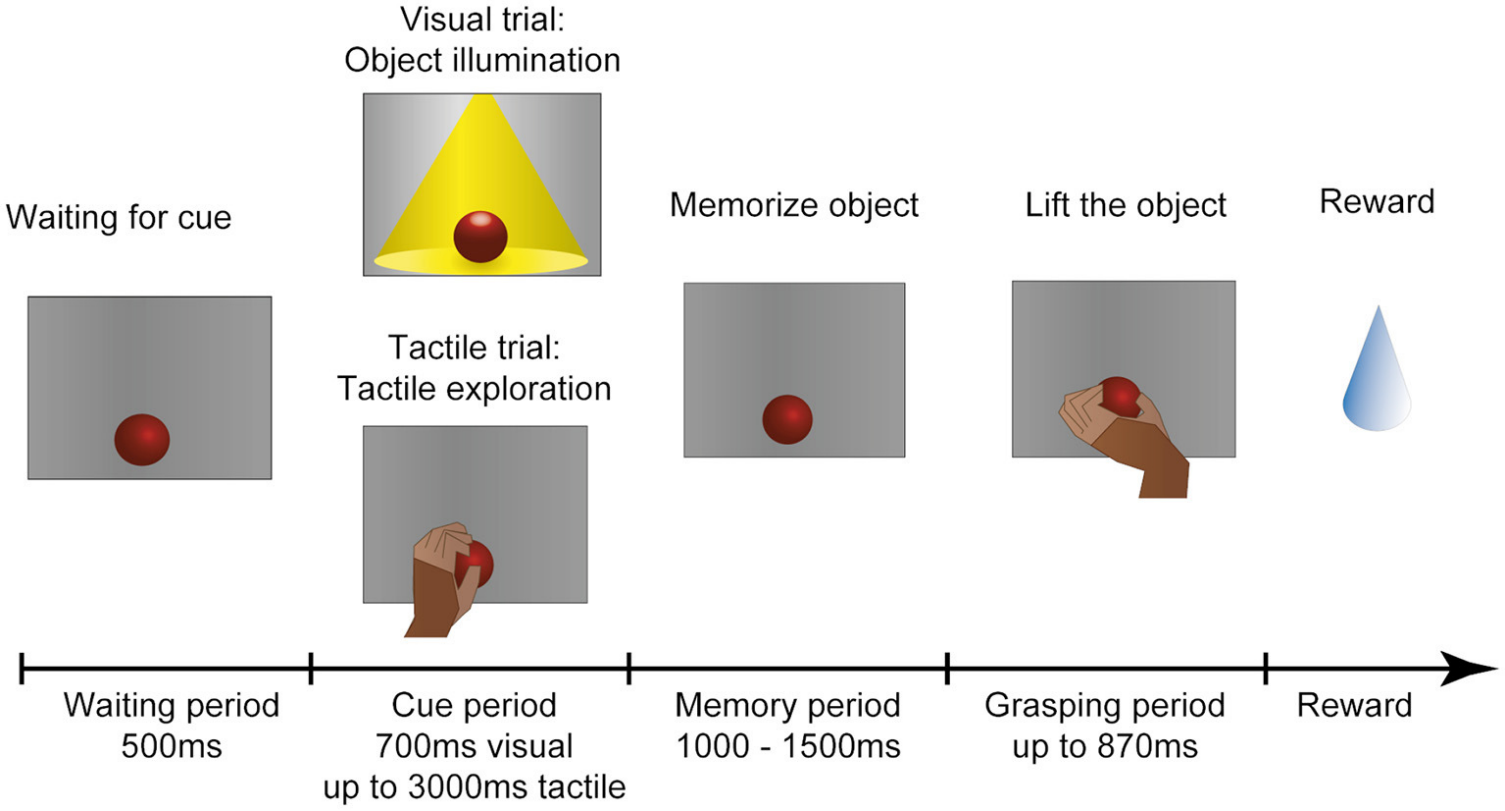
Task paradigm. After an object has arrived in front of the animal, he has to wait in the dark with the hand in a resting position. In the following cue period the animal could then identify the object either visually or tactually. In the visual task, the object is illuminated for 700 ms. In the tactile task (indicated to the animal by a red LED that turns off), the monkey remains in the dark and instead has to reach out, touch and briefly lift the object (maximal duration: 3,000 ms). The animal is then required to return his hand to the resting position and memorize the object for 1,000–1,500 ms before the red cue LED turns off and he is instructed to lift the object within 870 ms. All successful trials are rewarded with a fluid reward. First published in Buchwald et al. (2021).

The monkey was placed in a dark setup so that no other objects could distract the monkey. For an illustration of the task paradigm see Figure 4. At the start of each trial the motor would rotate the turntable and bring a random object to the front.

The task consisted of five epochs: First 500 ms of baseline epoch where recorded, where the animal had to sit still in the dark. Then the cue epoch followed. During visual trials the object was illuminated for 700 ms, so that the monkey could see the object. He was not allowed to touch it at this point. During tactile trials the opposite was true: A red LED instructed the animal to reach out in the dark and touch the object. To ensure that he indeed interacted with the object, he had to briefly lift it up. This assured that the whole hand of the monkey had contact with the object. Usually he explored it shortly, until he recognized the correct grip type to apply to this object. He was given 3,000 ms to reach out, explore and lift, and return his hand to the handrest position. Afterward the memory period followed, where the monkey had to memorize the object in the dark while keeping his hand placed on the handrest position. The duration of the memory period varied between 1,000 and 1,500 ms (chosen at random) to ensure that he could not predict the following go cue, in which the red LED turned off to instruct the animal to grasp the object in the dark. The movement epoch was kept short (870 ms) to ensure that the animal could not use this time epoch to further explore the object, but had to be ready to quickly select an appropriate grip type and lift up the object (for 700 ms) before returning to the handrest button in order to receive a fluid reward [small amount of his favorite juice (grape or pear)].

### 2.5. Data Analysis

#### 2.5.1. Movement and Reaction Time Analysis

The first important question was to check whether the animal was using the object information in this task, or whether he was simply able to guess a correct grip type, even when he could not know the object. For this, we compared the (pooled) reaction and movement times of all ten sessions in the various task conditions and modalities. Incorrect trials (such as failure to lift the object or trying to grasp during the memory period) were excluded from further analysis. Reaction time was defined as the time between the occurrence of the Go cue and the movement start. Movement time started when the animal lifted his hand from the handrest button and ended when the object was lifted completely. Results were plotted as a histogram and the mean reaction and movement times were calculated.

#### 2.5.2. Hand Kinematic Analysis

The second essential question was whether the animal would use the same grip type between visual and tactile trials. For this, the animal was equipped with the magnetic data glove that recorded the positions of seven sensors with up to 100 Hz. The position of these sensors could then be used to calculate the angles between each joint of the monkey's hand, as described in Schaffelhofer and Scherberger (2012). This data was then aligned with the behavioral task events to find where each trial started, ended and when the object interaction occurred. To check whether grip types between different modalities were similar, a linear discriminant analysis (LDA) was used and verified with leave-one-out analysis. This analysis searched for common features across trials that can be used to classify the data. Results were plotted in a confusion matrix. The goal was to test whether a classifier could differentiate between visual and tactile trials on the basis of hand kinematics, or gets confused between them. Since the number of trials per object varied between 14 and 18, a correction was done before each training step, so that the classifier used the same number of trials for each condition for training, avoiding an over-representation of some conditions. In addition to the classification approach, we also calculated the Euclidean distance between the grasp kinematics and displayed the clusters in a dendrogram.

#### 2.5.3. Neural Data Analysis

Neural data was recorded during ten session from four brain areas: AIP, F5, M1, and S1. During recordings, continuous data (30 kS/s) was collected and stored on a hard drive. Channels containing only noise signals were marked and later removed from further analyses. For spike detection, data was filtered with a median filter (window length: 3.33 ms), the resulting signal subtracted from the raw signal, and a 4th order non-causal Butterworth filter (5,000 Hz) applied to low-pass filter the data (Butterworth, 1930). Then, a principal component analysis (PCA) artefact cancellation procedure was performed, as described in Musial et al. (2002). This step was done to exclude common noise that occurred across channels, therefore only PCA dimensions with a coefficient larger than 0.36 (with respect to normalized data) were kept. For spike sorting, a modified version of Wave_Clus was used (based on Kraskov et al., 2004; Chaure et al., 2018). Furthermore, units with a firing rate below 1 Hz were excluded from all analyses. The number of remaining units for each recording session and area is provided in Table 1.

**Table 1:** Number of units with a firing rate higher than 1 Hz per area and recording day.

**Recording day**	**#units F5**	**#units AIP**	**#units M1**	**#units S1**
18.05.2018	36	5	88	60
01.08.2018	26	7	62	50
02.08.2018	27	6	54	46
07.08.2018	25	14	60	53
09.08.2018	24	14	55	52
04.06.2019	22	5	61	49
16.10.2019	22	7	69	61
24.10.2019	22	12	65	45
15.11.2019	28	9	67	43
22.11.2019	26	6	67	41

##### 2.5.3.1. Population Analysis

The spike sorted data was then further analyzed with a sliding window analysis of variance (ANOVA; window size: 100 ms) that investigated differences between the mean of groups of data points, here the number of spikes between the different conditions (objects) and modalities (visual and tactile trials). Afterwards a correction for multiple comparisons was applied (Bonferroni correction). The percentage of tuned units (y-axis) was then plotted over time (x-axis).

##### 2.5.3.2. Classification

To further quantify whether or not observed significant differences might be big enough for the brain to differentiate between the origin of sensory data (or simply between sensory modalities) a classifier was trained on the neural data. Again, LDA served as a basis for this step and the classifier was then validated using leave-one-out cross-validation. Here, a correction for the number of trials was implemented to ensure that no condition is over-represented by randomly selecting an equal number of trials for all conditions and modalities. Results are then plotted as a confusion matrix for three different time epochs: Early memory, late memory (first and last 500 ms of the memory period, respectively) and grasp epoch.

## 3. Results and Discussion

### 3.1. Movement and Reaction Time Analysis

**Figure 5:**
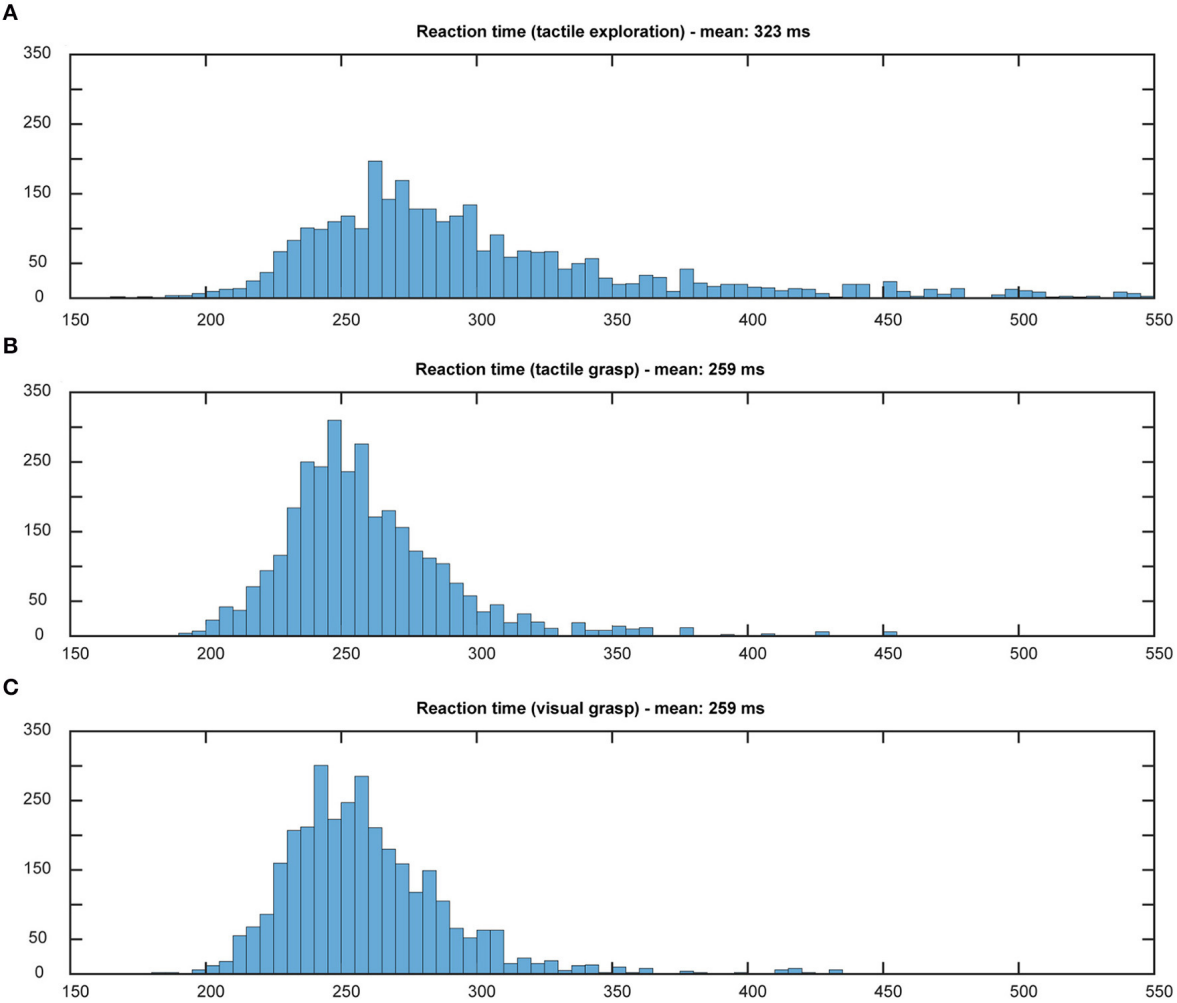
Distribution of reaction times. Histograms illustrate the distribution of reaction time in both tasks, all 10 sessions pooled. Bin width: 5 ms, cut off at 550 ms. During tactile exploration **(A)** the object was unknown and on average a longer reaction time could be observed. When looking at grasp movement execution in the tactile **(B)** and visual grasp **(C)** both distributions appear identical and with the same mean, indicating less hesitation due to knowledge about the object and therefore appropriate grasp to lift it.

**Figure 6:**
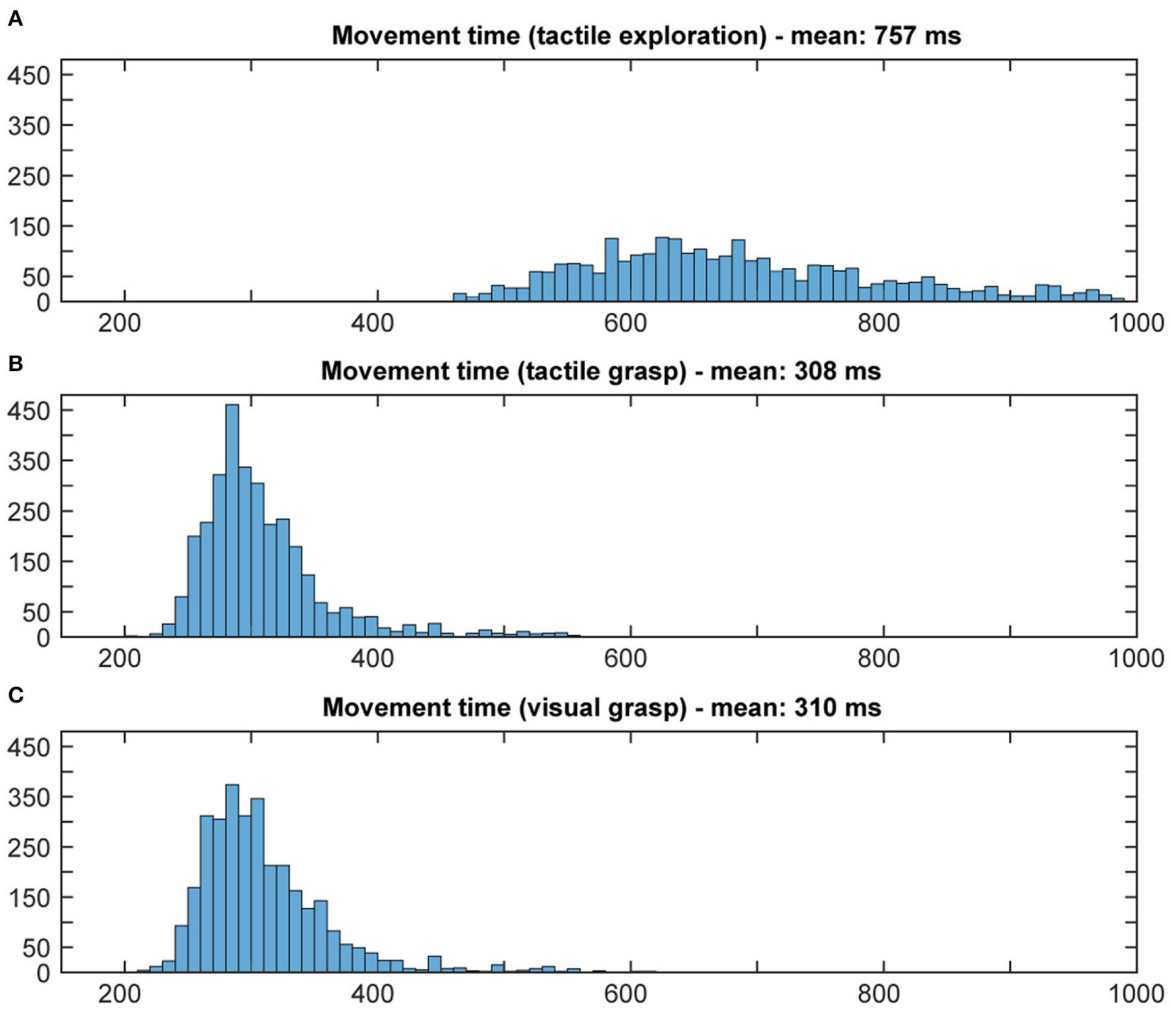
Distribution of movement times. Histograms illustrate the distribution of movement time in both tasks, all 10 sessions pooled. Bin width: 10 ms, cut off at 1,000 ms. During tactile exploration **(A)** the object was unknown, leading to multiple and varying grasp attempts and on average a much longer movement time. When looking at grasp movement execution in the tactile **(B)** and visual grasp **(C)** both distributions look similar, indicating that the animal was able to execute an appropriate grasp of the object based on the object information gathered from the cue period.

Using the above methods, we first tried to evaluate whether the animal would use the sensory information he collected during the cue period. The assumption is that grasping a known object will result in less hesitation, i.e., shorter reaction time, and faster movement times, especially when the animal is extremely familiar with the objects (Gibson, 1958; Eimas, 1967; Dhawan et al., 2019). Results can be found in Figures 5, 6. During tactile exploration (Figures 5A, 6A), a very broad distribution of longer reaction and movement times can be observed, with a mean of 323 and 757 ms, respectively. In Figures 5B,C, 6B,C, reaction and movement times are displayed during visual and tactile grasp period, respectively. In contrast, during movement execution, we found a very similar distribution of reaction time, with an identical mean of 259 ms, and likewise of movement time, with a mean of 308 and 310 ms for visually and tactually guided grasps, respectively. This indicates that the animal is aware of the object identity in both tasks. While this is somewhat obvious for visual trials, where the monkey saw the object and one can therefore assume that he goes the easier route of remembering the object and recalling the appropriate grip type that proved best in the past, this is also likely the case for tactile trials after tactile exploration. Touching the object and quickly lifting it up was sufficient to inform the monkey about the object identity, in line with results from human participants (Klatzky and Lederman, 1992). From observation and kinematic data we can also confirm that the monkey had to correct his hand shape in many trials during tactile exploration, since he usually approached quickly with a more uniform hand shape and only on touch found the correct grip (see example video in the Supplementary Materials). Not knowing the object therefore results in a longer movement time, further indicating that the shorter movement time during tactile grasp period stems from knowledge about the object information.

### 3.2. Hand Kinematic Analysis

An important check of behavior was to see whether or not the monkey grasps objects always with the same grip type. While monkeys generally optimize their grasping strategy to act as quickly as possible, it is not a priori clear how the animal weights different strategies. On the one hand, humans only need to make contact with a few key features of objects to correctly group them (Klatzky and Lederman, 1992). However, the animal might simply go with its first point of contact, which might lead to a more awkward but still successful lift as it depends on where the animal first hit the objects and managed to get a firm grasp. Here it probably helps that the monkey was trained for over a year before the first data was collected, and became very good at quickly distinguishing the six objects, similar to what was found in other animals (Gibson, 1958; Eimas, 1967; Dhawan et al., 2019).

**Figure 7:**
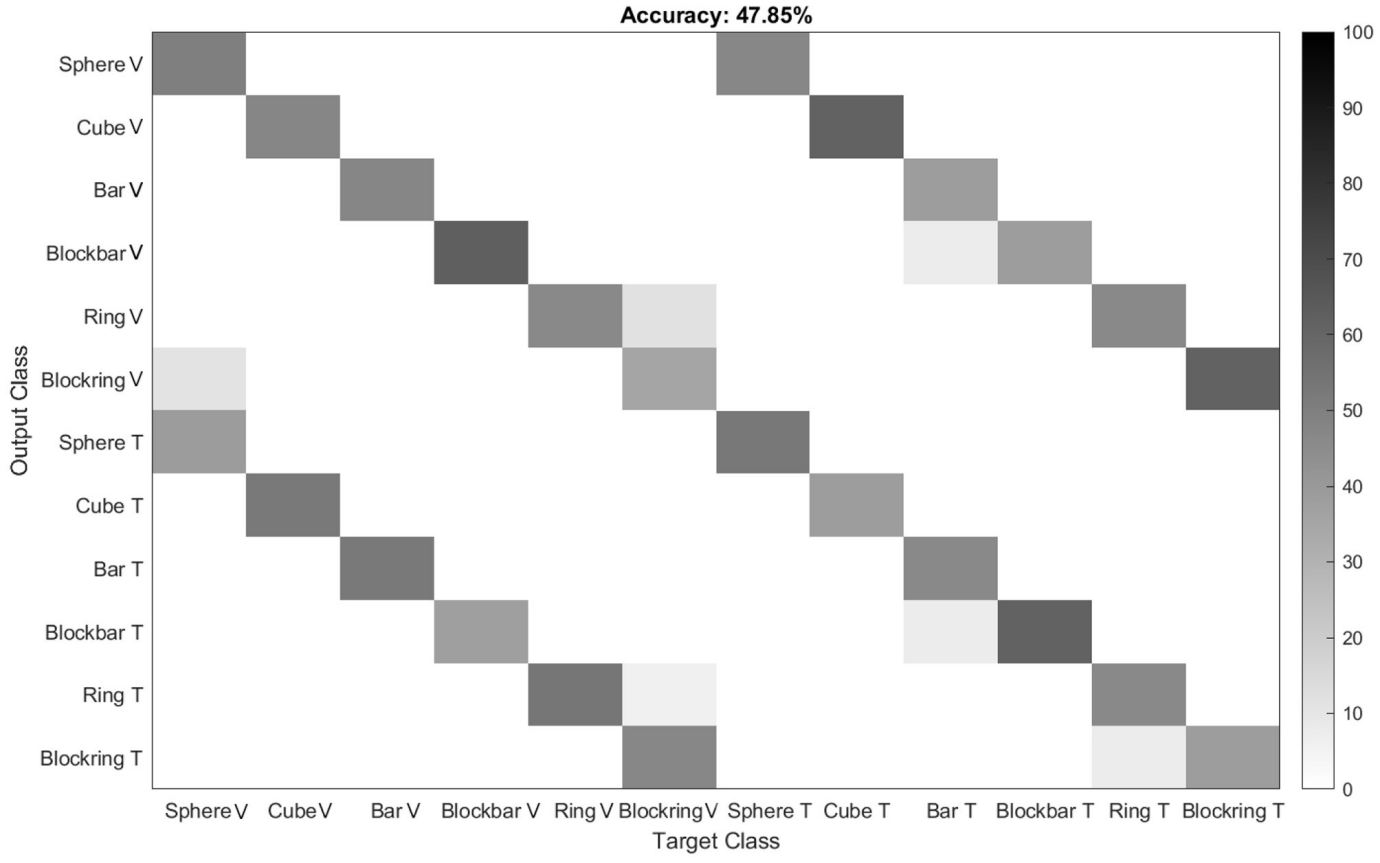
Classification of grip types from one example session. To evaluate whether the animal grasps the same object with the same grip during the visual and tactile task, an LDA classifier was trained on the kinematic data. Results indicate that the classifier indeed can not distinguish between visually and tactually guided grasps, with performance being close to chance level. Gray levels indicate classification performance in percent.

**Figure 8:**
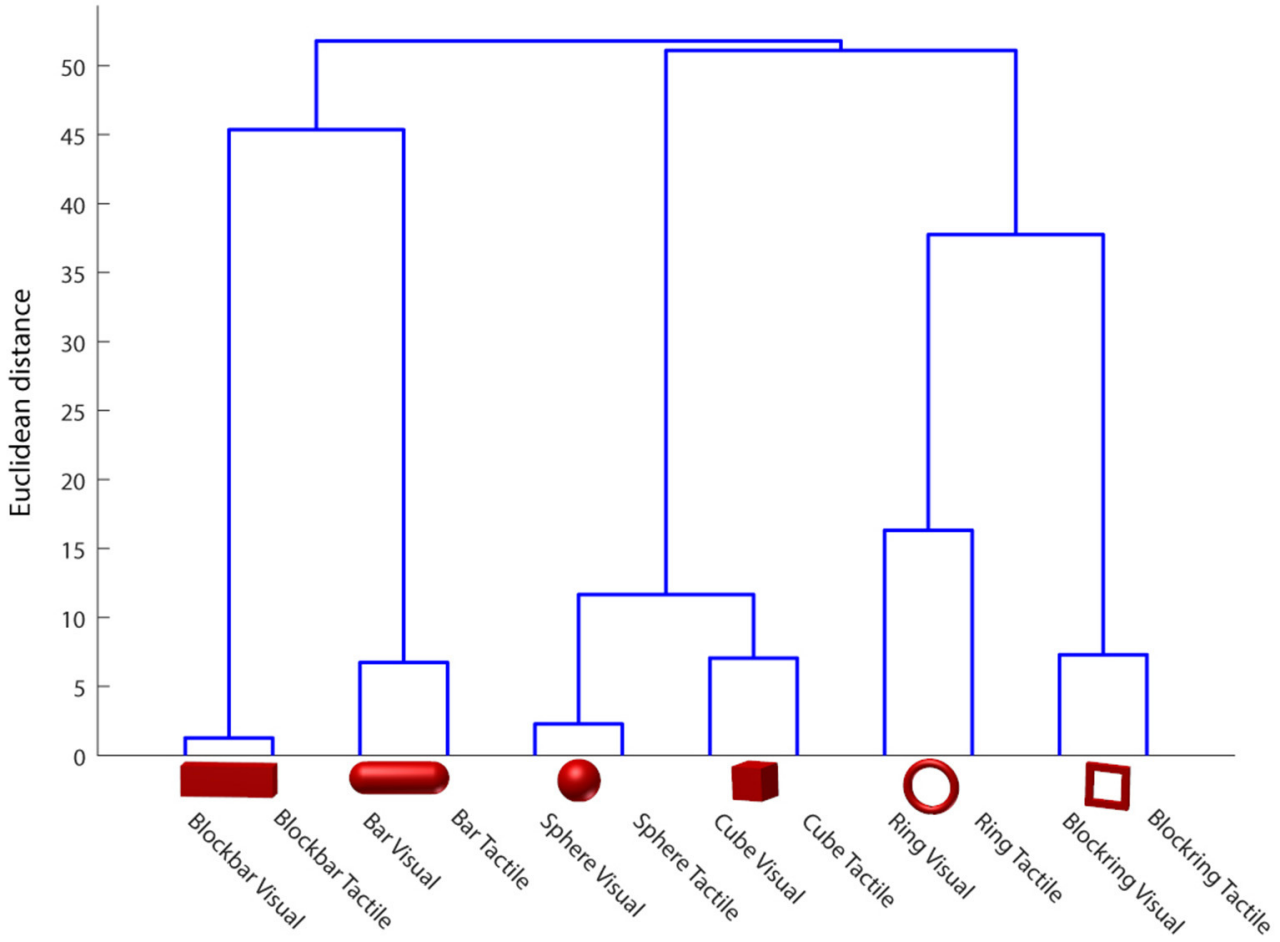
Hand shape difference based on Euclidean distance in the joint angle space. Euclidean distance between classes was calculated and plotted as a dendrogram, indicating high similarity between visually and tactually guided grasps to the same object. Data of one example session.

To test whether or not the monkeys uses the first or second strategy, we monitored the animal's hand kinematics via video, which revealed that the monkey has developed a dedicated grasp for each object that was applied independently of the sensory modality in a particular trial. Even during the tactile exploration phase, he quickly made contact with the object and after realizing which of the six objects he handled, adjusted his hand to his preferred grip type for that object (see video in Supplementary Materials). To quantify these observations, data from the magnetic kinematic glove was used to train an LDA classifier in the different modalities of this task and to evaluate the data using leave-one-out cross-validation (see Figure 7, top). To do so, we took the average joint angles between 450 and 550 ms after the animal successfully lifted the object. This ensured that the animal had already settled in a stable grasp position for each object and would no longer make grasp adjustments, but also would not yet loosen his grasp and return to the handrest button; this was allowed only after 750 ms after he had lifted the object. During this period, almost no confusion occurred between different objects, as became apparent by the three diagonals in the confusion matrix (Figure 7). For example, the condition “cube, visible” was only confused with “cube, tactile,” indicating that while the animal chose different grasps for all six objects, the sensory modality did hardly affect the chosen grip. This was somewhat remarkable, since the objects were initially designed in pairs, where each pair (cube/sphere, bar/blockbar, ring/blockring) was supposed to be grasped by the same grip type. While it was hard to recognize any grip differences between these object pairs by visual inspection, the kinematics analysis revealed that the animal did grasp each of the six objects at least slightly differently. One likely explanation is that while the objects were similar in size (with the diameter of all object pairs being identical), handling of round and edged objects was different, possibly to avoid object edges or simply because flat surfaces required slightly different finger positions or pressure distributions than round ones. The overall accuracy of the classifier was close to chance level (about 48%), hinting that while the object was easy to classify, the sensory modality had only a minor impact on the hand shape of the monkey. If the classifier was trained only on objects, independent of sensory modality, accuracy was about 93%. In contrast, if only the sensory modality was known to the classifier, it fell to chance level (52%), supporting the idea that while objects can be separated nicely (due to very distinct grasp movements), the sensory modality can not be decoded from the hand kinematics alone. This matched the observation that objects are grasped the same between sensory modalities and this was also a strong indicator that the differences in neural activity, as described below, are not driven by differences in grip types, but indicate differential neural processing due to different sensory input. Furthermore we clustered the joint angles by calculating the Euclidean distance between all grasps to see how much the grasp kinematics differed between sensory modalities (see Figure 8). We always found very low Euclidean distances for trials of different sensory modality (visual vs. tactual) and interaction with the same object.

### 3.3. Neural Population Analysis

**Figure 9:**
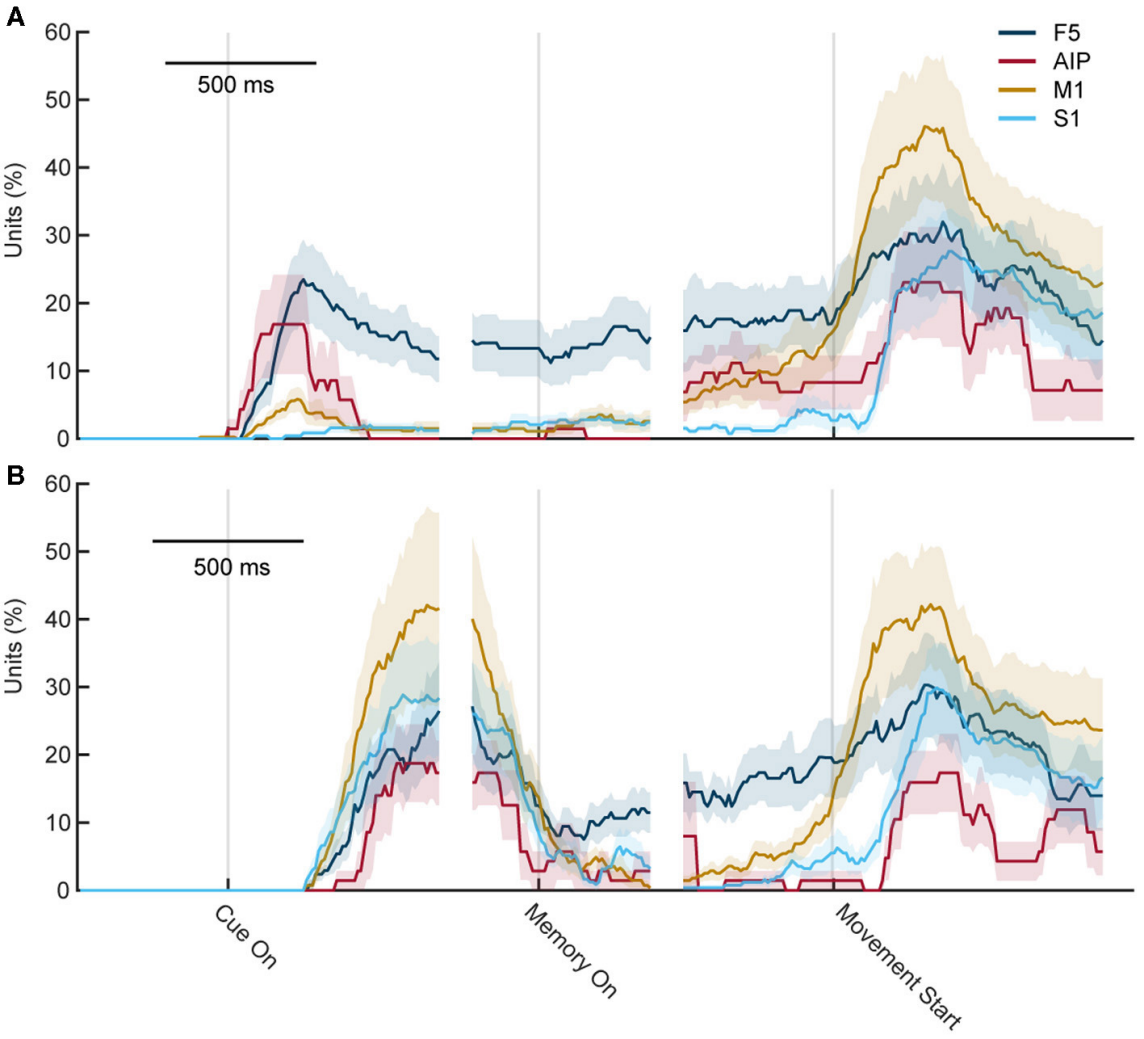
Percentage of object-selective units throughout both tasks. Percentage of units with significant selectivity for objects (ANOVA, factor objects) in the cortical areas F5, M1, AIP, and S1 for each time point during the task. **(A)** During visual trials, differences first rise when the object is seen and some remaining selectivity can be seen in F5. Shortly before grasping, object selectivity rises again and reaches a peak after movement start. **(B)** During tactile trials, the first rise is slightly delayed as the first 250 ms contains the reach movement of the exploration phase. F5 also shows some selectivity during movement planning and the main peaks occur during movement execution.

Neural data was recorded in parallel from 256 electrodes, four cortical areas, and across ten recording sessions. A sliding two-way ANOVA (factor sensory modality, two levels, and factor objects, six levels) was applied to test for significant differences between objects and sensory modalities in various epochs of the task. Figure 9 shows the fraction of neurons with significant selectivity for the six objects, separately for visual trials (Figure 9A) and tactile trials (Figure 9B). The overall result was similar for both trials, with the largest peaks of selectivity occurring when the object was interacted with, either when looking at it (mainly in F5 and AIP) or when it was tactually explored and lifted up. Furthermore, F5 showed sustained preparatory activity during the visual and tactile task, while this was much rarer in the other three areas. Only before movement start, the percentage of object-modulated neurons increased once again, with a peak during object interaction.

**Figure 10:**
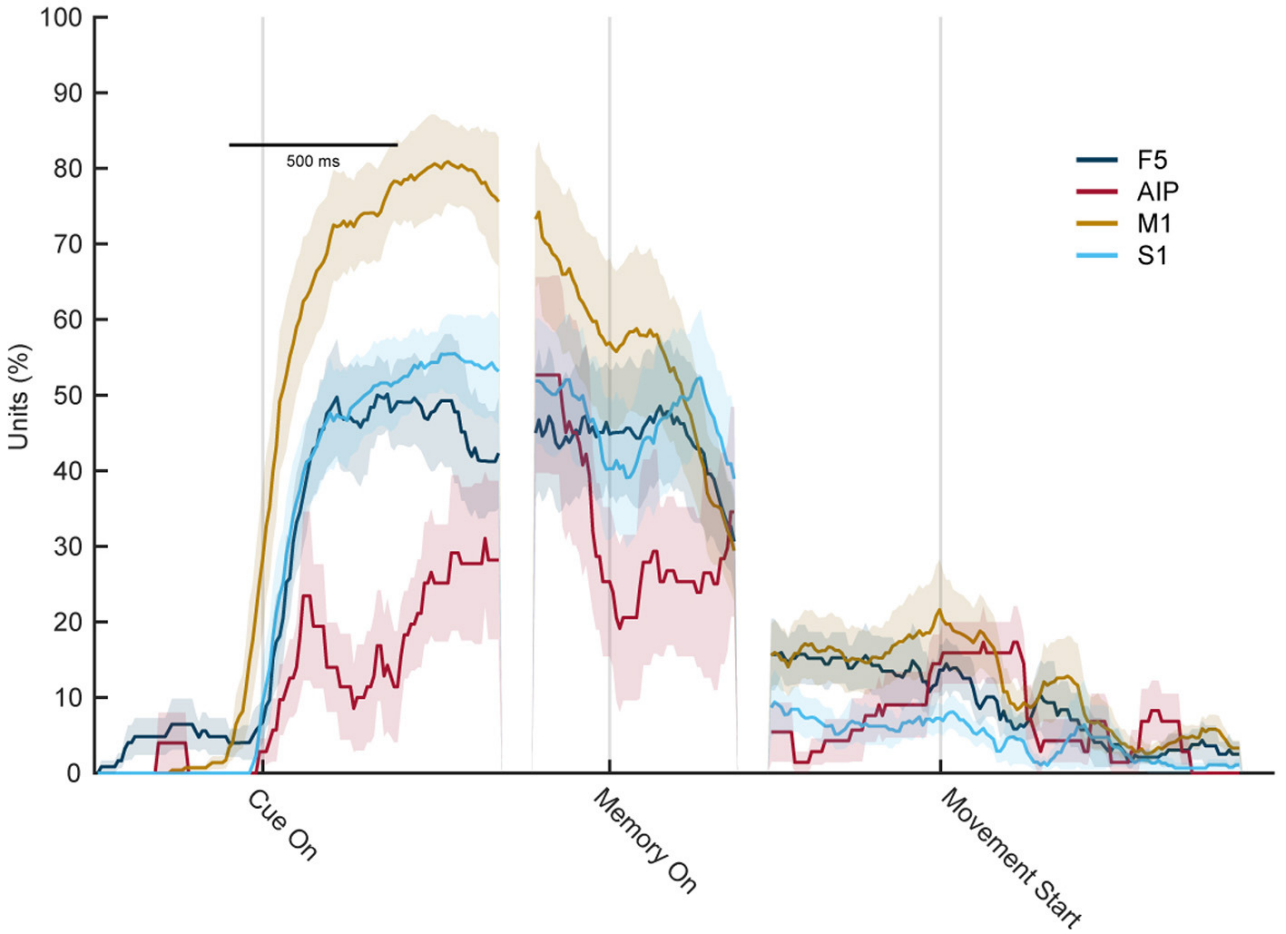
Percentage of sensory modality-selective units throughout the task. Percentage of units with significant selectivity for the sensory modality (ANOVA, factor sensory condition) in each area F5, M1, AIP, and S1 for each time point during the task. Early in the memory epoch, all four cortial areas show significant selectivity. However, this strongly decreases during the late memory and movement execution period, indicating a substantially reduced capacity of individual units to distinguish the sensory modality (vision vs. touch) at the time of movement execution.

Figure 10 shows the percentage of significantly selective units for sensory modality over time. High rates of selectivity were expected during the cue epoch, since the monkey performs two different actions (looking at an illuminated object while sitting still, vs. touching an object in the dark). In line with this setting, a high number of significantly tuned units was also observed at the start of the memory period, which is the time point when the light turns off or when the monkey returned to the handrest button after tactile exploration. Since he just stopped moving, a high number of significantly tuned units was expected. Interestingly, though, while this effect declined over time, it was still present in all four cortical areas shortly after movement start. While the effect was not as huge as during the early memory epoch, a number of units continued to show significant tuning even after movement start. Only about 250 ms after the movement started, the percentage of tuned units became insignificant for AIP and S1, whereas for M1 and F5, this downward trend took about 500 ms. This came as a surprise because as was established before, the movement the monkey executed during visual and tactile trials did not differ, at least not to an extent that a different hand shape could be observed or a classifier was able to decode the sensory condition from the hand shape. Therefore, even though the same movement was prepared in visual and tactile trials, a significant tuning differences was observed in the population analysis, which, however, might be based on a small pool of neurons.

### 3.4. Classification of Neural Data

**Figure 11:**
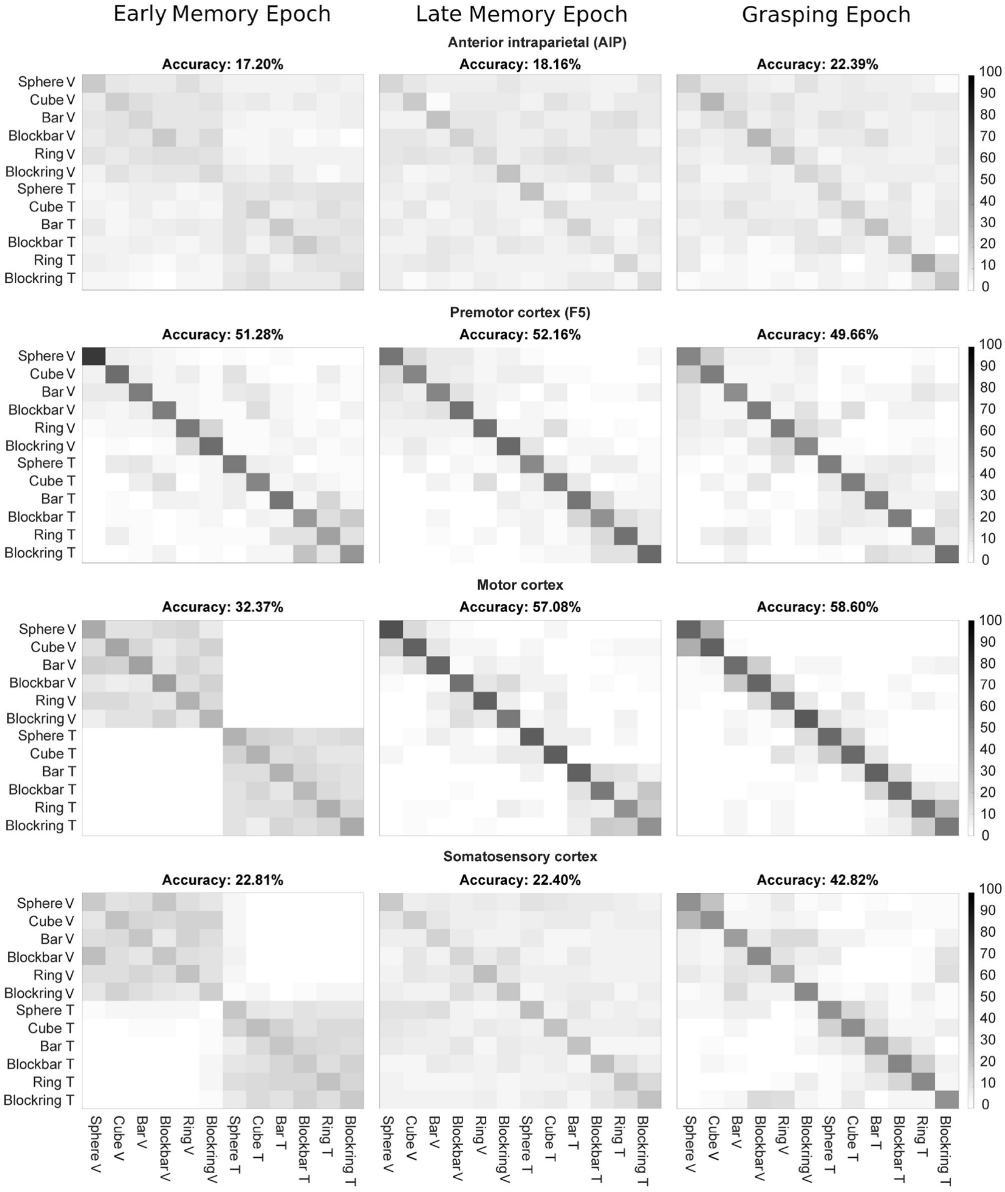
LDA decoding accuracy per epoch, averaged over five recordings. Confusion matrices showing decoding accuracy of 500 ms for three epochs (early memory, late memory, and grasping) for F5, M1, and S1. Decoded epochs are early memory, late memory, and grasping. Axis labels indicate the object in visual (V) and tactile (T) trials. X-axis: instructed task condition, y-axis: decoded task condition.

While the differences found by the neural population analysis may seem small, we wanted to evaluate whether they are strong enough for a classifier to recognize the sensory modality. For this, the neural data of each session was used to train an LDA classifier, validated with leave-one-out cross validation (see Figure 11). The resulting confusion matrix could show two distinct patterns: First, whenever the actual condition and prediction match, a diagonal should form in the middle of the confusion matrix. Secondly, since we first listed all visually and then all tactually perceived object conditions, a partition into four quadrants would indicate a separation between both sensory modalities, independent of objects. Chance level for classifying these 12 classes is 8.33% (six objects times two sensory modalities).

In AIP, which is known to react to the visual properties of objects about to be grasped, the overall performance of the classifier was low, even though above chance (17, 18, and 22% for Early, Late Memory and Grasping epoch, respectively). When only the sensory modality was decoded, a higher accuracy was reached during Early Memory (76%), likely due to a lasting influence of the visual activity during the visual task. This fell to 64% during Late Memory and 58% during Grasp. Similarly, due to the overall low performance of AIP neurons, decoding of object information from AIP was hard. During early memory, an accuracy of 22% was observed (chance level: 16.66; classification of six objects). Accuracy remained poor during late memory (27%) and only got better in the Grasping Epoch (36%) when tactile object interactions occurred. The overall low performance of object classification could be due to the low number of recorded units in AIP (5–14 units per session, see Table 1). It is also possible that the few recorded units were not object-selective, which would explain the mismatch of this study with the previous literature, e.g., see (Schaffelhofer and Scherberger, 2016). Still, one can see a stronger performance in decoding sensory modality in the early memory period, which once again might be a result of the two different tasks the monkey solved ahead of this period (seeing an object vs. touching an object in the dark). During late memory and grasping, classification of sensory modalities was harder and the main diagonal, indicating correct decoding, became spurious.

A stronger performance was observed in the premotor area F5 (20–36 units per session), where even during early memory a good decoding above chance level was found (51%), with a strong focus on determining not only the correct object (again 51% if only objects were classified), but also the correct sensory modality under which an object was perceived (92% when only sensory modality is decoded).

Areas M1 and S1 (54–80 and 41–61 units per session, respectively) also showed a similar pattern: During early memory, when the cue period still had a strong influence on the brain state (such as remaining motor activity only slowly receding) the sensory modality could be predicted well (the plots are separated into the four blocks where the visual and tactile objects are listed, 32% for M1, 23% for S1). For M1, when only objects were decoded the classifier reached an accuracy of 30% while sensory modality could be classified with 99% accuracy. Similarly, in S1 an accuracy of 21% for objects and 97% for sensory modality were found. This was followed by a good prediction of the object, but worse predictions of the sensory modality during late memory and grasping. This effect was stronger for M1, where the classification of objects reached 76% during late memory and 91% during grasping, when motor cortex was involved in leading the monkey's hand movements. For the sensory modality, accuracy fell from 78% in late memory to 64% during grasping, possibly because, at this point, movement execution (grasping according to object shape) became more important than the sensory origin of the object shape information. Similar findings could be seen in S1, although weaker. For object decoding, accuracy rose from 21% and 37% during early and late memory to 78%, possibly due to the direct influence of tactile perception on the brain area. On the other hand, classification accuracy of the sensory modality fell from 97% and 63 to 56%, close to chance level, similarly highlighting the fact that, while sensory modality still influences the brain activity during early memory, it becomes less important as the task progresses toward movement execution.

Overall these results indicate that activity in all four cortical areas can be used to predict the sensory modality under which an object was perceived at all three time points: best during early memory and late memory and with a performance close to chance level during grasping, whereas the additional classification of objects emerged particularly during late memory and grasp. These findings may not appear very surprising during early memory, however, it could have been expected that the different sensory modalities might completely disappear once they become irrelevant to the animal: shortly before the go cue appears, the monkey is most likely just focused on getting the grip type right, to earn his reward as quickly as possible, without unnecessary delays. At a cognitive level, it should therefore be irrelevant for the animal, how he learned about the object identity. Furthermore, the resulting movement is the same, whether the grip type was selected based on visual or tacile object information. This suggests that the same movement was either planned differently by the involved brain areas or these brain areas nevertheless maintain information about the underlying sensory modality, even after it has become irrelevant to the animal in the particular task.

## 4. Conclusion

In this study, we trained one rhesus monkey to grasp objects that he either saw or touched beforehand with the goal to determine whether or not the different sensory information would influence cortical grasp planning. We first could demonstrate that the animal used the same grip type for the same object, independent of the sensory modality of a trial. This was an interesting insight into the strategy used by the animal. Instead of going for an approach of not trying to recognize the identity of an object by thoroughly exploring it with his hand, which might require less effort, he not only explored the object sufficiently, but also maintained this information in order to perform an optimal grip type. For this, it is probably relevant that the animal was very familiar with the objects and obtained a high task performance (over 90% correct trials) throughout all sessions (Kaeser et al., 2014). Due to his familiarity with the objects, the monkey did not actually have to touch the whole object in front of him to recognize the object identity. Instead, it is likely that once his hand encountered one of the unique features of the objects (round vs. edge shape, thickness or others), he could recognize the object already. This was also confirmed by an analysis of the movement times. In analogy to visual trials, where the animals visually explored and recognized each object, a comparison to the same epoch in tactile trials showed that the animal had a similar movement time for both modalities (Camponogara and Volcic, 2021). However, during tactile exploration, his movements were slower, due to the need to frequently correct his handshape.

After having confirmed that any differences should not be a result of the monkey simply executing different movements in both tasks, we looked at the population activity of the four brain areas: AIP, F5, S1, and M1. Here, we found a small but significant difference during the movement planning. While the majority of recorded units did not actually show a significantly difference in their activity, it is interesting that a small subset either still encoded the sensory modality or any associated information, even though this should be irrelevant after the object was recognized and the grip type determined.

These finding have interesting implications for the classification of brain activity. Whereas the classifier should be able to predict the intended grip type independent of the sensory modality, completely ignoring this information could introduce noise, since the variance of neural activity rises. For practical applications, like the decoding of intended hand movements for neural prosthetics, one might therefore have to pay attention to the sensory modalities from which object information was acquired, since they might influence how the brain acts before and even after the start of the movement, even though the particular sensory modality might be irrelevant for the actual movement.

## Data Availability Statement

The original contributions presented in the study are included in the article/Supplementary Material, further inquiries can be directed to the corresponding author/s.

## Ethics Statement

The animal study was reviewed and approved by the Animal Welfare Division of the Office for Consumer Protection and Food Safety of the State of Lower Saxony, Germany (permit no. 14/1442 and 19/3132).

## Author Contributions

DB and HS conceived and designed the experiments. DB performed the experiments. DB analyzed the results. DB and HS wrote the article. All authors provided comments and approved the manuscript.

## Conflict of Interest

The authors declare that the research was conducted in the absence of any commercial or financial relationships that could be construed as a potential conflict of interest.
