# Supplementary material for: Visually and Tactually Guided Grasps Lead to Different Neuronal Activity in Non-human Primates
Source: Front Neurosci. 2021 Jul 19;15:679910. doi: 10.3389/fnins.2021.679910 (PMC8326571; doi:10.3389/fnins.2021.679910)
Supplement: Supplementary file 2 [file Data_Sheet_1.PDF]

Supplementary Material

1 SUPPLEMENTARY TABLES AND FIGURES

1.1 Figures

Figure S1. Supplemental figure 1: Video of the monkey performing a grasp movement. The animal is sitting in the dark, in front of the turntable and interacts with objects using his left hand. Text in the lower left corner indicates whether a trial is tactually or visually guided. At the start of each trial, the monkey places his hand on the handrest button until a cue LED (not in the frame) allows him to reach out and either tactually explore or lift and grasp the object.
